# Impact of intimate partner violence on *in vitro* fertilization outcomes among infertile women: a retrospective cohort study

**DOI:** 10.3389/fmed.2026.1914158

**Published:** 2026-09-08

**Authors:** Xuran Wang, Hongwei Yan, Cui Li, Yuhuan Feng, Shang Gao, Jiameng Zhang, Haiyan Zhao, Jinghua Zhang, Chang Guo, Mingjia Zhao

**Affiliations:** 1 North China University of Science and Technology School of Clinical Medicine, Tangshan, Hebei, China; 2 Tangshan Key Laboratory of Reproductive Medicine, Tangshan Maternity and Child Health Care Hospital, Tangshan, Hebei, China; 3 Tangshan Maternity and Child Health Care Hospital, Tangshan, Hebei, China

**Keywords:** *in vitro* fertilization-embryo transfer, infertility, intimate partner violence, mental health, miscarriage, pregnancy outcomes

## Abstract

**Background:**

Intimate partner violence (IPV) is a major public health concern that disproportionately affects women, yet its impact on assisted reproductive outcomes remains insufficiently understood. This study aimed to assess the 12-month prevalence of IPV among infertile women and to evaluate its association with *in vitro* fertilization–embryo transfer (IVF-ET) outcomes.

**Methods:**

This retrospective cohort study included 406 infertile women who underwent IPV assessment at a tertiary reproductive center in China between January 2020 and December 2022. IPV exposure within the previous 12 months and psychological status were assessed at baseline before embryo transfer using validated questionnaires. Among them, 325 women who underwent IVF-ET cycles were included in the analysis of reproductive outcomes. IVF-ET characteristics and pregnancy outcomes were collected from electronic medical records and follow-up data. Multivariable logistic regression models were used to evaluate the associations between IPV exposure and miscarriage and live birth.

**Results:**

The 12-month prevalence of IPV was 57.4%, with psychological violence being the most common form. Women exposed to IPV had poorer psychological health and a longer duration of infertility. IPV exposure showed a marginal association with an increased risk of miscarriage (adjusted OR 2.96, 95% CI 0.97–9.04; *P* = 0.057) and was independently associated with a reduced likelihood of live birth (adjusted OR 0.59, 95% CI 0.36–0.98; *P* = 0.041).

**Conclusion:**

IPV is highly prevalent among infertile women. It is independently associated with reduced live birth rate following IVF-ET, and shows a marginal association with increased miscarriage risk. Incorporating IPV screening into reproductive healthcare may help identify affected women and provide opportunities for appropriate support, potentially improving their psychosocial well-being and reproductive care.

## 1 Introduction

Infertility affects approximately 15%–20% of reproductive-age couples worldwide and imposes substantial psychological, social, and economic burdens (1). Although *in vitro* fertilization–embryo transfer (IVF-ET) is an established treatment for infertility, treatment success may be influenced by psychosocial and environmental factors in addition to biological determinants.

Intimate partner violence (IPV), including physical, psychological, sexual, and economic abuse, is a major threat to women's health and an important driver of health inequities (2). Women with infertility appear to be at greater risk of IPV than the general population, possibly because of social stigma, reproductive pressure, and gender norms surrounding fertility (3). Notably, a systematic review published in *The Lancet Global Health* reported that nearly half of women with infertility experience IPV during their lifetime, underscoring the seriousness and urgency of this public health issue (4).

Emerging evidence suggests that IPV may adversely affect reproductive health through multiple pathways. Chronic psychological stress may disrupt the hypothalamic–pituitary–ovarian axis, impair ovarian function, and reduce endometrial receptivity (5). Inflammatory activation and immune dysregulation may further compromise embryo implantation (6). Additionally, economic control and psychological distress may hinder access to IVF-ET and reduce treatment adherence, thereby contributing to poor outcomes (7). However, IPV remains insufficiently recognized in routine fertility care, leaving a gap between medical treatment and psychosocial support (8).

Despite increasing recognition of these mechanisms, existing studies have primarily focused on the prevalence and psychosocial consequences of IPV among infertile women. Recent qualitative evidence from women undergoing ART treatment suggests that infertility-related stress and reproductive pressure may contribute to psychological abuse, reproductive coercion, and partner control during fertility treatment (9). Moreover, population-based evidence indicates that IPV exposure is associated with adverse reproductive outcomes, including miscarriage (10). However, whether IPV exposure influences IVF-ET clinical outcomes, particularly miscarriage and live birth, remains insufficiently understood.

Therefore, this study aimed to investigate the prevalence of 12-month IPV among infertile women and its association with IVF-ET outcomes. We hypothesized that IPV exposure would be associated with adverse IVF-ET outcomes, including higher miscarriage risk and lower live birth rates.

## 2 Materials and methods

### 2.1 Study design and participants

This retrospective cohort study was conducted among infertile women at Tangshan Maternal and Child Health Care Hospital between January 2020 and December 2022. IPV exposure was assessed cross-sectionally at baseline before embryo transfer using a self-reported questionnaire. Participants were subsequently followed through IVF-ET treatment until reproductive outcomes, including miscarriage and live birth, were determined based on medical records and follow-up data.

Eligible participants were women aged 24–40 years who met clinical indications for IVF-ET and provided informed consent. Exclusion criteria included incomplete questionnaires, treatment at other institutions, or severe systemic or psychiatric conditions.

**Figure 1:**
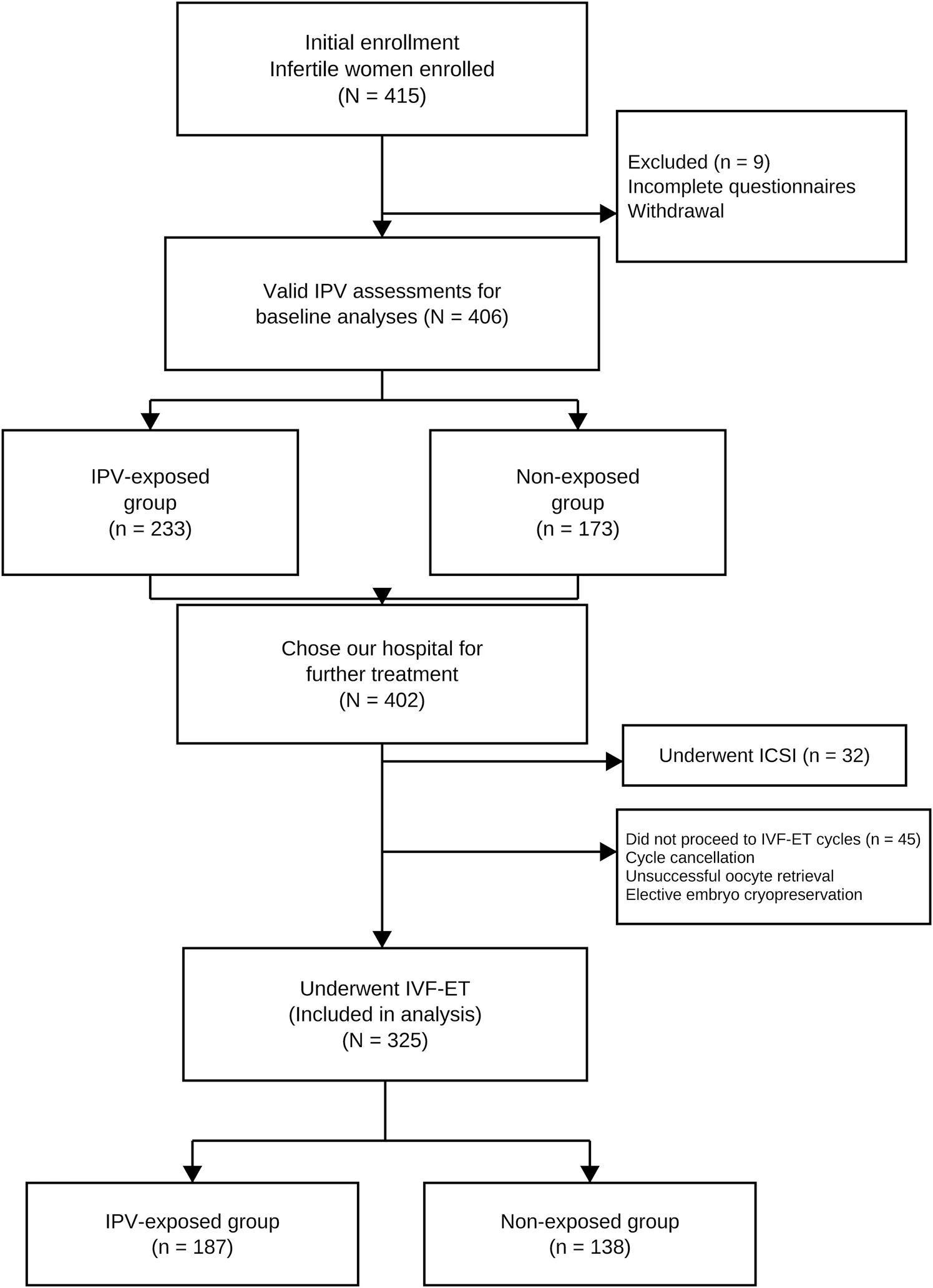
Flow diagram of participant enrollment, IPV assessment, and inclusion in the IVF-ET outcome analysis.

A total of 415 infertile women were initially enrolled. Nine participants were excluded due to incomplete questionnaires or withdrawal, leaving 406 women with valid IPV assessments for baseline analyses. According to self-reported IPV exposure within the previous 12 months, participants were categorized into the IPV-exposed group (*n* = 233) and non-exposed group (*n* = 173). Among these participants, 402 women selected our hospital for further treatment. Thirty-two women underwent intracytoplasmic sperm injection (ICSI), and 45 women did not proceed to IVF-ET cycles due to reasons including cycle cancellation, unsuccessful oocyte retrieval, or elective embryo cryopreservation. Finally, 325 women underwent IVF-ET and were included in the analysis of IVF-related clinical characteristics and reproductive outcomes, including 187 women in the IPV-exposed group and 138 women in the non-exposed group (Figure 1**)**.

### 2.2 Assessment of IPV and psychological status

IPV exposure within the past 12 months was assessed using a structured questionnaire incorporating the Revised Conflict Tactics Scales (CTS-2) and the Scale of Economic Abuse-12 (SEA-12), covering physical, psychological, sexual, and economic violence. The general CTS-2 items have previously been applied in the Third Survey on the Social Status of Women in China, supporting their use in Chinese populations. In the present study, the negotiation subscale was excluded, the assault and injury subscales were combined into a physical IPV domain, and the response categories of the SEA-12 were aligned with those of the CTS-2. Physical IPV was assessed using 18 items, psychological IPV using 8 items, sexual IPV using 7 items, and economic IPV using 12 items. Physical, psychological, and sexual IPV were further categorized as minor or severe according to the risk of injury or trauma requiring medical intervention (11). The measurement system showed acceptable psychometric performance in this population, with a test–retest reliability of 0.72, a Cronbach's *α* of 0.96, and acceptable model fit in confirmatory factor analysis (CFI = 0.80, TLI = 0.79) (12).

Psychological status was assessed using the Self-Reporting Questionnaire-20 (SRQ-20), a validated tool recommended by the World Health Organization. The SRQ-20 consists of 20 dichotomous items covering somatic, emotional, and cognitive symptoms, as well as suicide risk. Each “yes” response was scored as 1 and each “no” response as 0, yielding a total score of 0–20, with higher scores indicating poorer mental health. A positive response to item 17 was considered indicative of suicide risk.

### 2.3 Data collection and follow-up

IPV exposure was assessed at baseline after enrollment and before the occurrence of reproductive outcomes. Specifically, all participants completed the IPV questionnaire at their clinic visit during the IVF-ET treatment process, prior to embryo transfer and pregnancy confirmation. The questionnaire was administered by trained healthcare staff in a private consultation room without accompanying family members. Participants completed the questionnaire confidentially with one-to-one guidance, and completed questionnaires were reviewed on site to ensure data completeness. IVF-ET treatment characteristics and reproductive outcome data were obtained from the electronic medical record system. Participants were followed from embryo transfer until the final reproductive outcome was determined, including no clinical pregnancy, miscarriage, or live birth.

### 2.4 Outcome measures

The primary outcomes were miscarriage and live birth. Clinical pregnancy was defined as the presence of at least one intrauterine gestational sac on ultrasonography after embryo transfer. Miscarriage was defined as pregnancy loss after confirmation of clinical pregnancy and before fetal viability. Live birth was defined as the delivery of at least one living infant after IVF-ET.

### 2.5 Statistical analysis

Data were analyzed using SPSS version 27.0. Continuous variables were expressed as mean ± standard deviation (SD) and compared using the independent-samples t test when normally distributed. Non-normally distributed continuous variables were compared using the Mann–Whitney U test. Categorical variables were presented as frequencies and percentages and compared using the *χ*^2^ test. Effect sizes for between-group differences in psychological health scores were reported using Cohen's d. Between-group comparisons were considered exploratory, and *P* values were not adjusted for multiple comparisons.

Binary logistic regression analyses were conducted to examine the associations between intimate partner violence (IPV) exposure and reproductive outcomes, with miscarriage and live birth treated as separate dependent variables in distinct models. Variables eligible for multivariable adjustment were selected based on both statistical evidence and clinical relevance: factors that reached a significance threshold of *P* < 0.05 in univariable logistic regression, as well as variables with well-documented roles as confounders or predictors of reproductive outcomes in prior literature, were retained for further modeling. Maternal age was forced into all final models *a priori*, given its status as a well-established, strong predictor of both miscarriage and live birth and a core confounder in reproductive epidemiological research. In contrast, paternal age and other partner-related characteristics were excluded from the final models: their crude associations with study outcomes were substantially attenuated and lost statistical significance after adjustment for maternal age, and simultaneous inclusion of both partners’ ages would introduce unnecessary multicollinearity and compromise model stability. The final adjusted models accounted for IPV exposure, exposure to violence by others, maternal age, and key IVF-ET treatment parameters, including fresh versus thawed embryo transfer, number of embryos transferred, and embryo developmental stage. Adjusted odds ratios (aORs) with 95% confidence intervals (CIs) were computed to quantify the magnitude and precision of the identified associations.

## 3 Results

### 3.1 Participant characteristics

Compared with women without IPV exposure, IPV-exposed women were less likely to have a bachelor's degree or above, more likely to be self-employed, and more likely to report annual household income of 50,000–80,000 RMB, longer infertility duration, and infertility attributable to male factors (Table 1). With respect to partner characteristics, IPV exposure was associated with lower partner education, less stable employment, more frequent smoking and alcohol use, and a greater tendency for conflict with others (Table 2).

**Table 1:** Sociodemographic characteristics of infertile women.

Patients (*n* = 406)	Specific indicators	Non-exposed group (*n* = 173)		Exposed group (*n* = 233)		Test statistic *P value*
		*n*	%	*n*	%	
Age	≤30	48	27.7	46	19.7	x^2^ = 3.856
	31–35	76	43.9	108	46.4	*P* = 0.145
	>35	49	28.3	79	33.9	
Education	High school and below	64	37	97	41.6	x^2^ = 7.179
	Junior college	83	48	120	51.5	***P*** **=** **0.028**
	Bachelor's degree and above	26	15	16	6.9	
Employment	Employed (stable job)	84	48.6	78	33.5	x^2^ = 9.688
	Self-employed/Freelance	78	45.1	132	56.7	***P*** **=** **0.008**
	Unemployed	11	6.4	23	9.9	
Annual income	≤30,000 RMB	61	35.3	52	22.3	x^2^ = 10.76
	30,000–50,000 RMB	32	18.5	56	24	***P*** **=** **0.013**
	50,000–80,000 RMB	61	35.3	106	45.5	
	>80,000 RMB	19	11	19	8.2	
Number of miscarriages	None	91	53.2	118	50.6	x^2^ = 2.678
	1–2	70	40.9	108	46.4	*P* = 0.262
	3–4	10	5.8	7	3	
Sexual activity frequency	>7 times/week	1	0.6	1	0.4	x^2^ = 21.953
	4–7 times/week	15	8.8	0	0	***P* <.01**
	1–3 times/week	117	68.4	183	78.9	
	<1 time/month	38	22.2	48	20.7	
Infertility duration	≤2 years	59	34.5	56	24.5	x^2^ = 9.632
	3–5 years	80	46.8	103	45	***P*** **=** **0.022**
	6–8 years	20	11.7	37	16.2	
	>8 years	12	7	33	14.4	
Cause of infertility	Female	63	36.4	50	21.6	x^2^ = 11.732
	Male	32	18.5	51	22	***P*** **=** **0.008**
	Both	22	12.7	45	19.4	
	Unknown	56	32.4	86	37.1	
Treatment measures^a^	No	10	5.8	2	0.9	x^2^ = 8.386
	Yes	163	94.2	231	99.1	***P*** **=** **0.004**

a Treatment measures include *in vitro* fertilization-embryo transfer (IVF-ET), intracytoplasmic sperm injection (ICSI), and artificial insemination (AI).

**Table 2:** Sociodemographic characteristics of intimate partners of infertile women.

Patients (*n* = 406)	Specific indicators	Non-exposed group (*n* = 173)		Exposed group (*n* = 233)		Test statistic *P value*
		*n*	%	*n*	%	
**Patients**	**Specific indicators**	47	27.5	46	19.7	x^2^ = 4.031
Age	≤30	71	41.5	98	42.1	*P* = 0.133
	31–35	53	31	89	38.2	
	>35	52	30.0	81	34.8	x^2^ = 0.980
Education	High school and below	109	63	144	61.8	***P*** **=** **0.047**
	Junior college	20	11.6	13	5.6	
	Bachelor's degree and above	81	46.8	91	39.1	x^2^ = 10.316
Employment	Employed (stable job)	89	51.4	121	51.9	***P* <0.01**
	Self-employed	3	1.7	21	9	
	Unemployed or jobless	142	83	186	80.2	x^2^ = 0.535
Smoking frequency	Daily	23	13.3	50	21.5	***P*** **=** **0.015**
	1–2 times/week	12	6.9	24	10.3	
	1–3 times/month	2	1.2	8	3.4	
	<1 time/month	74	42.8	68	29.2	
	Never	61	35.3	111	47.6	
Drinking frequency	Daily	15	8.7	12	5.2	x^2^ = 13.208
	1–2 times/week	12	6.9	5	2.2	***P*** **=** **0.01**
	1–3 times/month	28	16.2	45	19.3	
	<1 time/month	57	33.0	60	25.8	
	Never	108	62.4	152	65.2	x^2^ = 12.925
Conflicts with others	Never	13	7.51	39	16.7	***P* <0.01**
	1–2 times	52	30.1	42	18	

### 3.2 Prevalence and patterns of IPV

Among the 406 infertile women, the 12-month prevalence of IPV was 57.4%. Psychological violence was the most common form (84.98%), followed by physical violence (64.80%), economic violence (52.36%), and sexual violence (16.31%). Common manifestations included belittling appearance, emotional coldness or silent treatment, insults or verbal abuse, pushing or shoving, financial monitoring, unilateral financial decision-making, and forced unprotected intercourse (Table 3).

**Table 3:** Distribution of violence-related data.

Type of IPV experienced	*n*	%^a^
Physical violence	151	64.80
Sexual violence	38	16.31
Emotional/psychological violence	198	84.98
Economic violence	122	52.36
Physical violence behaviors (*n* = 151)		
Throwing	49	32.45
Quarrel/fight (minor)	32	21.19
Shoving or pushing	78	51.66
Blow to the head causing unconsciousness	8	5.30
Sought medical treatment due to violence	26	17.22
Choking/strangulation	30	19.87
Spousal assault (severe)	19	12.58
Slapping	24	15.89
Burns or scalds	11	7.28
Sexual violence behaviors (*n* = 38)		
Coerced unprotected sexual activity	27	71.05
Violent coercion of atypical sexual acts	3	7.89
Atypical sexual acts against one's will	4	10.52
Violent forced intercourse	9	23.68
Intercourse against one's will	16	42.10
Emotional/psychological violence behaviors (*n* = 198)		
Insults or verbal abuse	102	51.51
Belittling appearance	171	86.36
Emotional coldness/silent treatment	113	57.07
Devaluing one's worth in the intimate relationship	69	34.85
Provoking/instigating anger	58	29.29
Verbal threats	46	23.23
Damage to personal belongings	66	33.33
Economic violence behaviors (*n* = 122)		
Appropriating essential living funds	48	39.34
Accumulating debt	54	44.26
Financial monitoring	83	68.03
Unilateral financial decision-making	72	59.02
Withholding information	57	46.72
Extreme financial control	38	31.15
Cutting off sources of income	31	25.41
Demanding that I resign	26	21.31
Using threats to prevent working	15	12.30

a Percentages of IPV subtypes were calculated among IPV-exposed women (*n* = 233). Percentages of specific behaviors were calculated within each IPV subtype.

### 3.3 Mental health status

Women exposed to IPV had significantly worse psychological health than non-exposed women. Mean total SRQ-20 scores were higher in the exposed group (5.81 ± 3.72 vs 3.35 ± 2.80, *P* < 0.001), and all symptom domain scores were elevated. Suicide risk was reported by 10.73% of IPV-exposed women compared with 1.16% of non-exposed women (*P* < 0.001) (Table 4).

**Table 4:** Between-group comparison of psychological health status.

Analysis dimension	Exposed group (*n* = 233)	Non-exposed group (*n* = 173)	Between-group difference test (U test) (Z value)	*P* value	Effect size (Cohen's d)^a^
Psychological health total score	5.81 ± 3.72	3.35 ± 2.80	−7.466	<0.001	0.75
Somatic symptom cluster	3.43 ± 1.93	2.29 ± 1.95	−5.823	<0.001	0.59
Emotional symptom cluster	1.55 ± 1.64	0.73 ± 1.10	−5.854	<0.001	0.59
Cognitive symptom cluster	0.73 ± 0.87	0.31 ± 0.64	−5.535	<0.001	0.55
Suicide risk item	10.73% (25/233)	1.16% (2/173)	X2 = 14.739	<0.001	

a Cohen's d was used to indicate the effect size for between-group differences in psychological health scores.

### 3.4 IVF-ET outcomes

The IPV-exposed group had a longer duration of infertility than the non-exposed group (3.17 ± 1.168 vs. 2.88 ± 1.134 years, *P* = 0.026). No significant between-group differences were observed in the number of embryo transfers, gestational age at delivery, or birth weight (Table 5). Variables considered clinically relevant or showing associations in univariable analyses were included in the multivariable logistic regression models. The results of univariable logistic regression analyses are presented in Supplementary Tables 1, 2.

**Table 5:** Comparison of IVF-ET pregnancy outcomes between the two groups.

Indicator	Non-exposed group	Exposed group	t/x2	*P*
Infertility duration	2.88 ± 1.134	3.17 ± 1.168	−2.234	0.026
Number of embryo transfers	1.46 ± 0.500	1.40 ± 0.491	0.998	0.319
Gestational weeks at delivery	37.286 ± 2.5696	37.843 ± 2.0667	−1.418	0.159
Birth weight	2,947.05 ± 688.324	3,058.17 ± 667.056	−1.043	0.299

Multivariable logistic regression analysis demonstrated a marginal association between IPV exposure and elevated miscarriage risk (Table 6). Women exposed to IPV tended to have higher odds of miscarriage relative to those without such exposure [adjusted odds ratio [aOR] 2.96, 95% confidence interval [CI] 0.97–9.04; *P* = 0.057]. No significant association was observed between exposure to violence by others and miscarriage (aOR 1.02, 95% CI 0.19–5.47; *P* = 0.983). After adjustment for confounding factors, none of the IVF-ET treatment parameters—including fresh versus thawed embryo transfer, number of embryos transferred, and embryo developmental stage at transfer—showed a statistically significant association with miscarriage risk.

**Table 6:** Multivariable logistic regression analysis of selected factors associated with miscarriage.

Variable^a^	Adjusted OR	95% CI	*P* value
IPV exposure (yes vs. no)	2.96	0.97–9.04	0.057
Violence by others (yes vs. no)	1.02	0.19–5.47	0.983
Women age (per year)	1.06	1.01–1.12	0.020
Fresh/thawed embryo transfer	1.28	0.42–3.92	0.668
Number of embryos transferred	1.36	0.50–3.73	0.548
Cleavage-stage embryo/blastocyst transfer	1.86	0.64–5.44	0.259

a Selected variables from the multivariable logistic regression model are shown. Adjusted OR, adjusted odds ratio; CI, confidence interval; IPV, intimate partner violence; IVF-ET, *in vitro* fertilization–embryo transfer.

In the multivariable model for live birth, IPV exposure was independently associated with a reduced likelihood of live birth (Table 7) (aOR 0.59, 95% CI 0.36–0.98; *P* = 0.041). Exposure to violence by others showed a borderline inverse association with live birth (aOR 0.41, 95% CI 0.17–1.01; *P* = 0.052). Fresh embryo transfer was associated with a markedly lower probability of live birth relative to thawed embryo transfer (aOR 0.24, 95% CI 0.14–0.40; *P* < 0.001), whereas transfer of a greater number of embryos was independently associated with increased odds of live birth (aOR 2.12, 95% CI 1.32–3.41; *P* = 0.002). Embryo developmental stage at transfer did not show a significant independent association with live birth (aOR 0.70, 95% CI 0.43–1.14; *P* = 0.151).

**Table 7:** Multivariable logistic regression analysis of selected factors associated with live birth.

Variable^a^	Adjusted OR	95% CI	*P* value
IPV exposure (yes vs. no)	0.59	0.36–0.98	0.041
Violence by others (yes vs. no)	0.41	0.17–1.01	0.052
Women age (per year)	0.90	0.87–0.93	<0.001
Fresh/thawed embryo transfer	0.24	0.14–0.40	<0.001
Number of embryos transferred	2.12	1.32–3.41	0.002
Cleavage-stage embryo/blastocyst transfer	0.70	0.43–1.14	0.151

a Selected variables from the multivariable logistic regression model are shown. Adjusted OR, adjusted odds ratio; CI, confidence interval; IPV, intimate partner violence; IVF-ET, *in vitro* fertilization–embryo transfer.

## 4 Discussion

In this study, our findings indicate that IPV exposure is independently associated with reduced live birth rate following IVF-ET, and shows a marginal association with increased miscarriage risk, collectively pointing to an adverse effect of IPV on reproductive outcomes. These findings complement evidence from a U.S. postpartum cohort, underscoring the complex relationship between infertility, fertility treatment, and IPV across populations and clinical contexts (13). Previous studies have mainly focused on the prevalence and psychosocial consequences of IPV among infertile women, whereas its impact on ART outcomes remains insufficiently understood. A recent qualitative study reported that women undergoing ART may experience psychological abuse, reproductive coercion, and treatment-related partner control, highlighting ART as a vulnerable period for women exposed to IPV (9). Moreover, a systematic review and meta-analysis demonstrated a high burden of IPV among infertile women and suggested that infertility-related stress may increase vulnerability within intimate relationships (4). Our findings further extend the literature by linking IPV exposure with clinically relevant IVF-ET outcomes and highlight the importance of considering IPV screening in fertility care settings. However, the association between IPV exposure and miscarriage should be interpreted with caution. Although IPV exposure showed a marginal association with an increased risk of miscarriage, the wide confidence interval suggests that the precision of the estimate was limited, possibly due to the relatively small number of miscarriage events. Therefore, this finding should be considered exploratory and requires confirmation in future studies with larger sample sizes and sufficient statistical power.

Several biological and psychosocial pathways may potentially explain the observed association between IPV and adverse IVF-ET outcomes. Chronic stress related to IPV exposure may influence neuroendocrine regulation and reproductive function, while stress-related inflammatory responses and immune alterations may also affect implantation and early pregnancy maintenance. In addition, IPV-related psychosocial factors, including reduced treatment adherence, limited access to healthcare, and financial constraints, may contribute to poorer reproductive outcomes (14–16). However, these mechanisms remain hypothetical and require further validation in future studies.

Women in the IPV-exposed group were less likely to have higher educational attainment and more likely to be self-employed, to report middle-range household income, and to have male-factor infertility. These characteristics may reflect limited social and economic resources and greater relationship strain, which could increase vulnerability to IPV (12, 17, 18). Husbands in the IPV-exposed group also tended to have lower educational levels, higher unemployment, more frequent smoking and alcohol use, and a greater tendency to engage in interpersonal conflict. This pattern is consistent with previous evidence from China showing that adverse partner characteristics are associated with a higher risk of IPV among women with infertility (11).

The 12-month prevalence of IPV in our cohort was 57.4%, which was higher than the pooled estimate reported for low- and middle-income countries, but lower than rates reported in Turkey, Iran, and Pakistan (19–21). Psychological violence was the most common subtype in this cohort, suggesting that humiliation, emotional withdrawal, and verbal abuse may represent the predominant forms of violence among women with infertility (22). Because psychological abuse is less visible than physical assault, it may remain undetected in reproductive clinics unless clinicians ask directly (20). Economic violence was also common, which may reflect the substantial financial burden of repeated IVF-ET treatment and its effect on household stress and conflict (23). The relatively high IPV prevalence observed in our study may be partly explained by the unique stressors experienced by women undergoing fertility treatment, including prolonged infertility duration, uncertainty regarding treatment outcomes, repeated treatment failures, and emotional distress. These factors may increase psychological vulnerability and interpersonal conflict among couples seeking ART.

IPV exposure was also associated with markedly worse mental health. Women in the IPV-exposed group had significantly higher mental health symptom scores and a 9.2-fold higher risk of suicide than those in the non-exposed group, indicating that IPV may substantially aggravate psychological distress among women with infertility (24, 25). These findings highlight the importance of integrating psychological screening and timely interventions, such as cognitive behavioral therapy, into fertility care for women exposed to IPV (26).

From a public health perspective, these findings support the integration of routine IPV screening into fertility care. Comprehensive interventions, including psychological support, couple counseling, partner involvement, legal education, and gender-transformative strategies (27–29), may help improve women's safety, reduce psychosocial distress, enhance treatment engagement, and mitigate the adverse effects of IPV on IVF-ET outcomes.

## 5 Limitations

This study has several limitations. First, although IPV exposure was assessed before embryo transfer, it was self-reported and may be subject to recall bias and underreporting, while changes in IPV exposure over time could not be fully captured. Therefore, causal interpretations should be made cautiously. Second, although several IVF-ET-related variables were adjusted for, some potential confounders were not systematically collected, including ovarian reserve indicators, number of retrieved oocytes, oocyte-related parameters, embryo quality, and detailed embryo transfer characteristics. These unmeasured factors may influence IVF-ET outcomes and the observed association between IPV exposure and reproductive outcomes. In addition, subgroup analyses of ovarian stimulation protocols were not feasible due to heterogeneity and uneven sample distribution. Future multicenter studies with comprehensive clinical and embryological data are warranted.

## 6 Conclusion

IPV exposure within the past 12 months was highly prevalent among infertile women undergoing IVF-ET. It was associated with poorer psychological health and a significantly reduced likelihood of live birth, and showed a marginal association with an increased risk of miscarriage. These findings suggest that IPV exposure may represent an important psychosocial factor associated with IVF-ET outcomes. Incorporating IPV screening and psychosocial assessment into fertility care may facilitate the identification of affected women and provide opportunities for appropriate support; however, further prospective studies are warranted to clarify the potential impact of IPV-related interventions on reproductive outcomes.

## Data Availability

The raw data supporting the conclusions of this article will be made available by the authors, without undue reservation.
