# Supplementary material for: Impact of intimate partner violence on in vitro fertilization outcomes among infertile women: a retrospective cohort study
Source: Front Med (Lausanne). 2026 Sep 8;13:1914158. doi: 10.3389/fmed.2026.1914158 (PMC13595577; doi:10.3389/fmed.2026.1914158)
Supplement: Supplementary file 1 [file Datasheet1.pdf]

Supplementary Table,

Table 1. Univariable logistic regression analysis for prespecified inclusion with miscarriage

Variable	Wald χ²	cOR	95% CI	*P* value
IPV exposure (ref: No IPV exposure)	4.56	3.01	1.09–8.27	0.033
Violence by others (ref: No violence by others)	0.04	0.85	0.19–3.81	0.835
Women age (per year)	7.83	1.07	1.02-1.13	0.005
Husband age (per year)	0.90	1.03	0.96-1.11	0.344
Fresh embryo transfer (ref: Thawed embryo transfer)	0.48	1.38	0.55–3.43	0.490
Number of embryos transferred (ref: 1 embryo)	0.12	1.16	0.50–2.67	0.729
Blastocyst transfer (ref: Cleavage-stage embryo)	1.01	1.53	0.67–3.53	0.315
Infertility duration (per year)	0.13	1.07	0.75–1.52	0.719
Partner conflict tendency (ref: No conflict tendency)	0.01	0.95	0.39–2.29	0.904

Note: cOR = crude odds ratio; SE = standard error. Binary variables are coded such that odds ratios represent the effect of the exposed/case group compared with the reference group. Continuous variables are modeled linearly, with odds ratios representing the effect per 1-unit increase. Variables were considered for multivariable modeling based on univariable associations (P < 0.05) and prespecified clinically relevant IVF-related covariates.

Table 2. Univariable logistic regression analysis for factors associated with live birth

Variable	Wald χ²	cOR	95% CI	*P* value
IPV exposure (ref: No IPV exposure)	8.02	0.52	0.33–0.82	0.005
Violence by others (ref: No violence by others)	5.47	0.35	0.15–0.85	0.019
Women age (per year)	42.67	0.90	0.87-0.93	<0.001
Husband age (per year)	11.19	0.93	0.89-0.97	0.001
Fresh embryo transfer	35.12	0.23	0.14–0.38	<0.001
Number of embryos transferred	12.25	2.23	1.42–3.51	<0.001
Blastocyst transfer	5.09	0.60	0.38–0.93	0.024
Infertility duration	0.24	0.95	0.79–1.15	0.627
Partner conflict tendency	2.90	0.66	0.42–1.06	0.088

Note: cOR = crude odds ratio; SE = standard error. Variables with P<0.05 in univariable analysis were considered for inclusion, in addition to a priori forced inclusion of key IVF treatment variables.
